# Anatidae Migration in the Western Palearctic and Spread of Highly Pathogenic Avian Influenza H5N1 Virus

**DOI:** 10.3201/eid1211.060223

**Published:** 2006-11

**Authors:** Marius Gilbert, Xiangming Xiao, Joseph Domenech, Juan Lubroth, Vincent Martin, Jan Slingenbergh

**Affiliations:** * Université Libre de Bruxelles, Brussels, Belgium;; † University of New Hampshire, Durham, New Hampshire, USA;; ‡ Food and Agriculture Organization of the United Nations, Rome, Italy

**Keywords:** Avian influenza, Epidemiology, Disease Ecology, Migration, perspective

## Abstract

During the second half of 2005, highly pathogenic avian influenza (HPAI) H5N1 virus spread rapidly from central Asia to eastern Europe. The relative roles of wild migratory birds and the poultry trade are still unclear, given that little is yet known about the range of virus hosts, precise movements of migratory birds, or routes of illegal poultry trade. We document and discuss the spread of the HPAI H5N1 virus in relation to species-specific flyways of Anatidae species (ducks, geese, and swans) and climate. We conclude that the spread of HPAI H5N1 virus from Russia and Kazakhstan to the Black Sea basin is consistent in space and time with the hypothesis that birds in the Anatidae family have seeded the virus along their autumn migration routes.

## Abstract 2

Anatids may have spread the virus along their autumn migration routes.

The spread of highly pathogenic avian influenza (HPAI) H5N1 virus during 2003–2004 in eastern and southeastern Asia, and, in 2005–2006 westward across Asia into Europe, the Middle East, and Africa is not typical of other HPAI epizootics. Until recent events, HPAI outbreaks or epizootics were assumed to first require transmission of a low pathogenic avian influenza (LPAI) virus from wild birds to domestic poultry (*1*). Preventive measures thus focused on surveillance and control in poultry and on stopping transmission to noninfected premises. Usually this strategy successfully extinguished an outbreak, often within the year (*2*). The spread of the disease back to wild birds from domestic fowl was considered relatively rare. The HPAI H5N1 virus is unusual in that virus infections in wild bird populations may cause a high proportion of deaths (*3**–**5*). A list of species that have been infected with HPAI H5N1 virus can be found in Table A1. The virus also is persisting in Asia longer than most previous HPAI epizootics, which suggests a local reservoir (*6**,**7*).

Epidemiologic studies during 2004–05 mainly focused on HPAI H5N1 virus persistence in relation to the agro-ecology of poultry and duck production systems (*6*), and little attention was paid to the role of wild birds in local viral persistence or long-distance spread during that period. Because of 3 major events during mid- to late 2005, wild birds are now suspected of spreading the HPAI H5N1 virus over long distances through migration (*8**,**9*). First, in May 2005, a major HPAI HN51 virus outbreak was discovered in wild birds in Lake Qinghai (western People's Republic of China), an important breeding place for migratory bird species in eastern Asia. Within a few weeks, several hundred birds, mainly bar-headed geese, had died of the disease (*4**,**10*). Eventually, other bird species also became affected, and several virus introductions may have occurred. Soon after the Lake Qinghai episode, HPAI H5N1 virus was detected in wild birds in Mongolia, to the north of Lake Qinghai along the central migration route, and in an area where domestic poultry were scarce (*11*). Second, in October 2005, HPAI H5N1 virus spread westward across Eurasia; outbreaks were recorded in Turkey, Romania, and Ukraine, usually in or near known wintering sites for migratory birds. This long-distance spread took place through areas with no record of any virus presence. Third, in spring 2006, the virus infected large numbers of mute swans and other wild bird species across western Europe, also in areas where no outbreaks had previously been detected in domestic poultry, despite continual and intensive surveillance. These incursions occurred after unusual waterfowl movements that were associated with a spell of cold weather in the Black Sea area where HPAI H5N1 virus is believed to have been endemic since autumn 2005.

Arguments may also be raised against the hypothesis that HPAI H5N1 virus is transmitted by wild birds. Invariably, wild birds found to be infected with the virus were either dead or moribund and may not have been able to spread the virus over long distances. Furthermore, in several cases, no straightforward match was found between the appearance of the virus and the presence of the wild birds suspected of spreading it. For example, HPAI H5N1 virus outbreaks that took place in Russia and Kazakhstan during summer 2005 were distributed along important trade routes that link western People's Republic of China to Russia (*12*), rather than any direct migration pathway.

We document and discuss the possible role of migratory birds in the spread of HPAI H5N1 virus during the second half of 2005, on the basis of information and data concerning the role of waterfowl in the ecology of avian influenza viruses; the pattern of Anatidae bird migrations across the western Palearctic, and contemporary, satellite-derived temperature data.

## Role of Migratory Waterfowl in Ecology of Avian Influenza Viruses

Avian influenza viruses (AIVs) have been recorded in most bird families (*3*), but the prevalence and diversity of AIV subtypes is not evenly distributed among them (*13*). AIVs have been isolated in 12 bird orders, but most isolations have been reported in the orders Anseriformes (in particular in the family Anatidae: ducks, swans, geese) and Charadriiformes (shore birds, gulls, terns). Although a wide variety of AIV subtypes have been isolated from Charadriiformes (*13**,**14*), they are believed to belong to a somewhat different genetic pool from those isolated in Anseriformes (*15*).

Species from the Anatidae family, in particular, the Anatinae subfamily (ducks), represent the highest risk for transmission to domestic poultry (*16**,**17*) for the following reasons: 1) Anatids harbor the most diverse and highest prevalence of avian influenza viruses (*13**,**14*); 2) historical outbreaks of HPAI in poultry have been linked mainly to strains circulating in ducks, rather than in members of other species (*18**,**19*); 3) domestic ducks (mallards) can excrete large amounts of HPAI H5N1 virus while remaining relatively healthy and are thus able to move the virus across large distances (*7*); and 4) direct contacts between wild anatids and domestic aquatic poultry are believed to be relatively more common than with other groups of wild birds (*20*).

Most waterfowl migrate seasonally, to exploit temporary feed resources during spring and summer, while escaping harsh winter conditions (*21*). An important evolutionary incentive for these migrations is the prolific spring growth in the Arctic, which provides plants and insects rich in the calcium and protein required for egg production (the female mallard duck produces 8–12 eggs) and juvenile growth (*22*). However, the favorable season for breeding is very short in these higher latitudes, and migratory bird populations soon start migrating southward with their juveniles to escape the frosts that occur from midsummer onward (*23*). This frequently results in premigration concentrations of many species of waterfowl south of the breeding areas, where juvenile maturation and adult molting take place before the main southward migration in the autumn. This seasonal aggregation mixes many species with high densities of immunologically naive juveniles alongside adult birds, which are unable to fly for up to 1 month while they molt; this setting is ideal for AIV transmission and redistribution. Previous work on AIV ecology has shown that premigration concentrations of waterfowl, together with the high recruitment rate of immunologically naive juveniles, induce a seasonally and geographically distinct pattern in AIV prevalence with peaks observed just before autumn migrations in interspecies concentration areas (*13**,**24*). During the subsequent southwest wards migration, AIV prevalence declines as a result of increased flock immunity and progressive dispersal of bird populations (*13**,**24**,**25*). Although AIV is more difficult to detect in waterfowl during winter and spring, several studies reviewed by Stallknecht and Shane (*13*) reported that AIV isolates persist until spring. De Marco et al. (*26*) demonstrated that AIV circulated continually from November to March in wintering areas. The high level of flock immunity and the relatively low level of AIV isolation during winter and spring raise the question of AIV survival during this time in the annual cycle (*13*). The survival of the virus in water and ice (*27*) may play a critical role, in terms of virus persistence and in terms of facilitating fecal-oral AIV transmission (*16*). The possible overwintering of AIV in shallow and cold water (*28*) in the Pan-Arctic region and the concentrations during postsummer-breeding and transmission of AIV between subpopulations and bird species during premigration may help sustain the natural AIV cycle. A redistribution of AIV among birds that use different migration routes may well contribute to the survival of the virus across a wide geographic range.

## Anatidae Migration Patterns in the Western Palearctic

**Figure 1:**
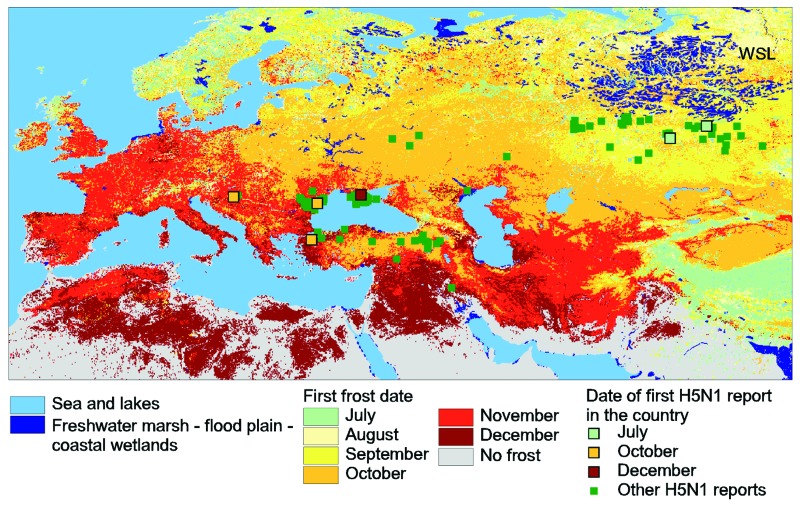
Map showing the spread of highly pathogenic avian influenza (HPAI) H5N1 virus and its environmental context. The background color indicates the month when the first frost was observed, from July through December 2005. The distribution of the main wetlands is indicated (dark blue; west Siberian lowland [WSL]). The reported presence of HPAI H5N1 virus from July 2005 to January 16, 2006, is indicated by squares with color coding for the first report of HPAI H5N1 virus in the country, and by green dots for other records.

**Figure 2:**
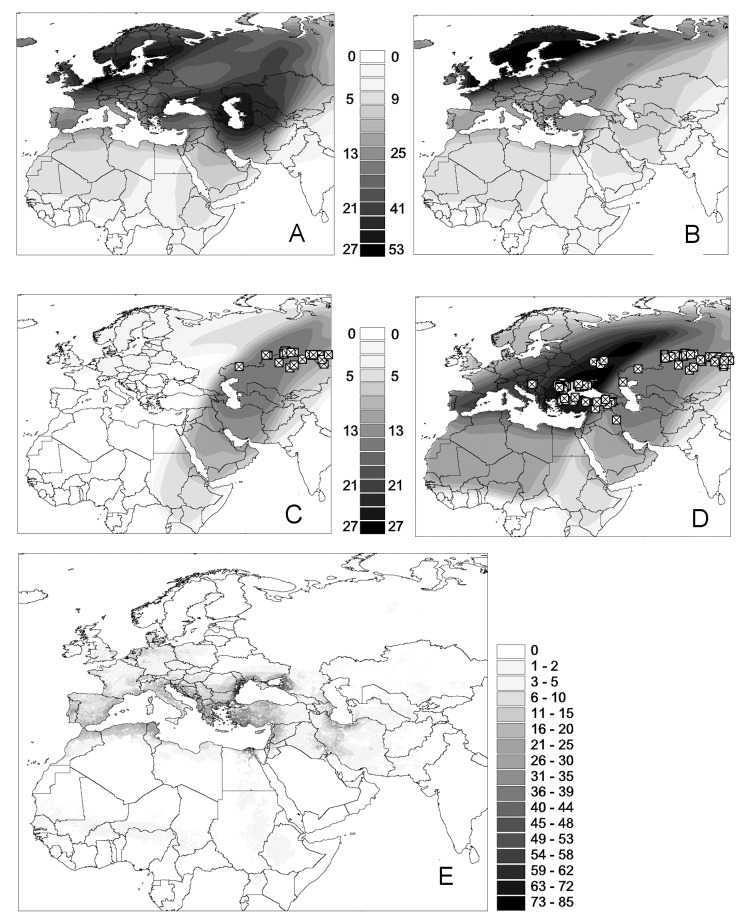
Distribution overlay of migratory flyways of Anatidae bird species in the western Palearctic: each pixel of gray shading indicates the number of species that include the area as part of their flyway. A) All species with an equal weight (indicative of species diversity by pixel). B) Flyways weighted according to their population (indicative of anatid populations). Population-weighted distribution overlay for flyways intersecting highly pathogenic avian influenza (HPAI) H5N1 virus records are shown for C) July through August and D) July through December. E) The maps displayed present the product of map B by the overlay of anatids wintering distribution and by the log_10_ of poultry density, as an index of the risk for transmission of HPAI H5N1 virus from anatids to domestic poultry in their wintering sites under the affected flyways.

**Figure A1:**
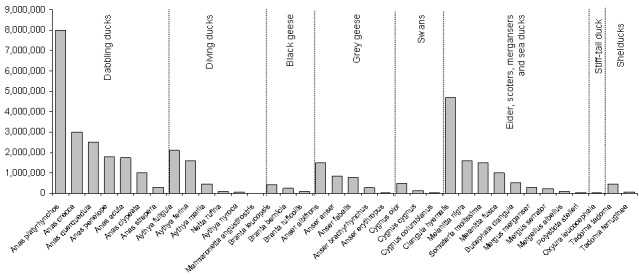
Population of Anatidae in the western Palearctic (*7*).

Northeastern Russia and Siberia are major breeding areas for many migratory Anatidae species in the Palearctic. Birds arrive during the spring, traveling different routes from Europe, Asia, and Africa. Of particular importance is the west Siberian lowlands (WSL), which has an area of 2,745,000 km^2^ and is by far the largest wetlands in the world (Figure 1). WSL is an important breeding area, along with several other large wetlands located in northwestern Russia and northern Scandinavia (Figure 1). In western Europe, the main wetlands that support wintering waterfowl are found along the coastal areas of Denmark, the Netherlands, United Kingdom, France (the Rhône delta), Spain, and northern Italy. In central Europe and western Asia, major wetland areas are found around the Black Sea in Ukraine, Romania (the Danube delta), and Turkey; around the Caspian Sea in Russia and Iran; and in the southeastern part of Iraq. Three recognized routes, or flyways, connect breeding areas to wintering areas in the western Palearctic (*29*) are shown on the overlay of individual species flyways in Figure 2A. The North Sea flyway joins the wetlands of northwestern Russia to western Europe wintering sites and runs through Scandinavia, the Baltic basin, and the North Sea. The Black Sea and Caspian Sea flyways run from the WSL, leading to Mediterranean Europe and western Asia, respectively. When weighted according to the number of birds that use them (Figure 2B), the North Sea flyway stands out as the most important, followed by the Black Sea; the Caspian Sea flyway is of least consequence. (The estimated population of Anatidae in the western Palearctic is shown in the Figure A1.)

## Migration Patterns and Spread of HPAI H5N1 Virus across the Western Palearctic

During July and August 2005, several HPAI H5N1 virus outbreaks were reported in Russia and Kazakhstan (Figure 1). These occurred in domestic poultry, but the strains were genetically related to the Lake Qinghai strain (*30*). Early in October 2005, HPAI H5N1 virus was first encountered in wild fowl and in poultry in Turkey and Romania and in dead swans in Croatia. Again, the sequenced virus was found to be identical to that from Lake Qinghai. This same virus was detected in Ukraine in December 2005 (*31*).

A mission of the World Organization for Animal Health (OIE) to Kazakhstan established that the first outbreaks were located near important molting sites for migratory waterfowl. This finding is further illustrated in Figure 1, which depicts the WSL breeding area, where the first frost took place as early as July (the pale green pixels). The premigration concentration of wild bird populations for molting takes place just south of the WSL. HPAI H5N1 virus may have been brought into southern Siberia through the poultry trade and related traffic, in particular, through the trans-Siberian commercial route (*12*), and, from there, may have entered the wild bird population. However, the observed patterns of virus circulation are also consistent with the critical steps in AIV transmission and redistribution (*13*), and HPAI H5N1 virus may have already been present in the wild bird population during the 2005 spring season in the WSL or at the molting sites and transmitted from there to domestic poultry at the time of main virus transmission in the premigration concentration areas.

The initial outbreaks of HPAI H5N1 virus in Romania, Turkey, and Ukraine occurred close to wetlands frequented by overwintering migratory waterfowl. These locations were clearly far from any known location where HPAI H5N1 virus had been recorded, while the timing and location match the autumn wildfowl migration ahead of the approaching wave of frost (Figure 1).

Figure 2C illustrates the population-weighted overlay of all Anatidae species flyways that coincide with locations of HPAI H5N1 virus in Russia and Kazakhstan observed during the summer 2005, i.e., the spread to be expected if only the bird species using the Caspian Sea flyway had been responsible for further spread. Figure 2D shows the population-weighted overlay of species flyways in relation to locations of HPAI H5N1 virus until January 2006. This figure suggests that the Black Sea flyway was also infected if anatids did indeed spread HPAI H5N1 virus through their autumn migration. This apparent discrepancy between HPAI H5N1 virus outbreak locations (Figure 2D) and the pattern of spread that could have been expected from the summer outbreaks locations (Figure 2C) requires further attention. First, the northern limits of the 2 flyways are so close that a figure similar to Figure 2D is obtained just with the summer outbreaks if one simply allows for a 200-km variation in the flyway border or if HPAI H5N1 virus presence is assumed to have occurred 200 km further westward. Second, the boundaries between the flyways are arbitrary; these flyways mainly represent directions taken by subpopulations, representing most diffusive migration paths. A large fraction of wild bird populations distributed across the area with locations of HPAI H5N1 virus reported presence in Russia and Kazakhstan connect to the Black Sea basin (actually, the number of anatids flying from Siberia down to the Black Sea is higher than the number flying to the Caspian Sea basin; Figure 2B). Given the above uncertainties, one may reasonably assume that waterfowl from both flyways may have become infected when they met in premigration concentration areas.

If these flyways are assumed to be used by infected birds, their geographic extent may be used to estimate an index of risk for virus transmission from wildfowl to domestic poultry. The population-weighted overlay of individual waterfowl species' ranges intersecting with HPAI H5N1 virus locations (Figure 2B) can, in turn, be intersected with the wintering areas' boundaries (rescaled from 0 to 1 as an index of wintering site suitability) to delineate the areas where migrating birds are more likely to concentrate. This layer can then be multiplied by overall poultry population density (*32*) to derive a coarse measure of risk for an HPAI H5N1 virus outbreak to occur in domestic poultry (Figure 2E). The resulting display highlights the high-risk areas on the Black Sea coast, in particular, areas adjacent to the wintering areas in Romania, Turkey, and Russia, but also parts of Greece, along the Adriatic coast, the Nile delta, and along the southern edges of the Caspian Sea. A relatively high risk is found across western Europe, which reflects not only the high density of poultry but also the fact that a cluster of 3 westernmost outbreaks in Russia intersect with multiple western European flyways.

## Discussion

Our results indicate that the broad-scale pattern of spread of HPAI H5N1 virus from Russia to the Black Sea basin is consistent with the spatial and temporal pattern of Anatidae migration from Siberia. Given that the first recorded signs of HPAI H5N1 virus in Turkey, Romania, and Ukraine took place in the direct vicinity of important waterfowl overwintering sites, Anatidae could have been implicated in the spread of HPAI H5N1 virus to the Black Sea basin. The search for wild bird species carrying HPAI H5N1 virus is in progress and awaits further classification. Several species demonstrably carry the virus without showing clinical signs, as has been recently reported from studies in Russia (*30*) and People's Republic of China (*8*). Most wild birds found dead were geese, swans, and, rarely, wild ducks (when domestic ducks were found infected with, and sometimes dead from HPAI H5N1 virus, this occurred in conjunction with disease outbreaks in terrestrial poultry), which supports the hypothesis that not only mallards but also several other duck species are healthy carriers of HPAI H5N1 virus. The postulate that migratory anatids can spread the disease over long distances by no means excludes the role of the poultry trade as an important, complementary transmission pathway.

It could be argued that an important contradiction of the hypothesis that wild birds spread HPAI H5N1 virus along their migration paths stems from our "false-positive" predictions (e.g., Figure 2E, Spain, Morocco, Greece). We propose 3 possible explanations for these deviations. First, as well as being along flyways of infected wild bird, establishment of HPAI H5N1 virus in domestic poultry may require additional conditions: 1) an aggregation of waterfowl for a sufficient period (more risk for transmission within wintering areas than at more transient stopover sites), 2) a high proportion of small poultry farms and backyard poultry, and 3) extensive (aquatic) poultry units in contact with waterfowl populations and habitat, i.e., floodplain or other forms of wetland agriculture in close proximity to natural wetlands used as wildfowl wintering sites. Such conditions have been shown to be associated with HPAI H5N1 virus persistence in Southeast Asia (*6*) and were certainly also met in parts of Romania, Turkey, and Ukraine. Second, the overall prevalence of HPAI H5N1 virus found in wild bird populations was very low, usually <1% (*8*). This finding suggests that virus persistence in wild bird populations may be subject to stochastic fluctuation. Also, few infected individual birds are likely to be evenly distributed in the population; i.e., the distribution of infected birds is probably clustered. The scarcity of infected individual birds and their likely clustering produce a pattern in which several regions exposed to equivalent wintering populations may have been exposed to different levels of virus exposure. Finally, HPAI H5N1 virus was found either in dead and apparently healthy ducks, which suggests a dichotomy in wild bird susceptibility. The exact status of species, as sentinels or spreaders, and precise migratory pattern may help explain any inconsistencies that arise from considering all species at equal risk for transmission.

One could also mention here the discrepancies between the geographic spread of HPAI H5N1 virus and overall pattern of wild bird migrations: the virus has never been reported in the Philippines and in several countries farther south such as New Zealand and Australia (although these 2 countries have no migratory anatid populations connecting them to Southeast Asia, they do have many shore bird and wader species in common [20]). Conversely, with the possible exception of African countries, HPAI H5N1 virus was established in domestic poultry only in countries connected by flyways with existing infected countries. The introduction in Nigeria is inconclusive. Two species of dabbling ducks, Anas querquedula and A. acuta, have large wintering concentrations in and near Lake Chad and in the Niger delta, both under the western Siberia/Black Sea flyways, and are presumed to be infected by HPAI H5N1 virus. However, Nigeria imported large numbers of poultry from Turkey and People's Republic of China until a ban was imposed, and illegal trade may well have continued after the ban and brought in infected animals or products (*33*).

The broad approach adopted in this study has clear limitations, given the uncertainties regarding the host range of HPAI H5N1 virus within the Anatidae family, the sizes and distribution of the bird populations, their precise migratory patterns, and the demarcation of the summer and winter habitat. A comprehensive retrospective analysis of HPAI H5N1 virus spread in the western Palearctic would require a better description of the dynamic distribution of wild birds (breeding range, wintering sites, stopover sites, migration pathways) as well as more detailed domestic poultry data (distribution, production structure, species composition, movements through trade) to map the contact points between wild and domestic birds. In addition, local studies could focus on possible introduction points and characterize and detail the specific ecologic conditions in the wild birds–domestic poultry interface that support establishment of the virus, including the local landscape structure (wild bird habitat and farming), climate (e.g., virus survival in the environment), and other agro-ecologic conditions.

## Experimental Procedure

### Imagery

We used the land surface temperature (LST) data products derived from the Moderate Resolution Imaging Spectroradiometer (MODIS) sensor on board the National Aeronautics and Space Administration's Aqua satellite (*34*). The Aqua satellite acquires daytime images (a local pass time of 1:30 p.m. at the equator) and nighttime images (a local pass time of 1:30 a.m. at the equator). A day/night algorithm was applied to a pair of MODIS daytime and nighttime observations to extract average temperature (when multiple observations are available), and the method yields an accuracy of 1°K with known emissivities (*34*).

Daily LST products were aggregated (averaged) to generate 8-day composite LST product (MYD11A2), and 46 of these 8-day composite LST products are generated per year. The LST product has a spatial resolution of 1 km. We downloaded the 8-day composite LST data (MYD11A2) in 2005 from the US Geological Survey Earth Resources Observation and Science (EROS) Data Center. For each individual 1-km pixel, we analyzed time series data of nighttime LST in 2005 and identified the first 8-day period that experienced frost (LST <0°C) in the fall/winter seasons. We assume that the date of early frost events in fall/winter seasons is one of many factors that affect the starting date of wild bird migration from north to south.

### Distribution Overlays

Distribution data regarding the winter feeding areas and summer breeding areas were extracted from the Global Registry of Migratory Species CD-ROM (*35*). The data on migration flyways were digitized directly from Scott and Rose (*36*). All boundaries were smoothed by a 2.5-decimal degrees filter to avoid sharp edges in distribution boundaries. The population estimates from Delany and Scott (*37*) were assigned to each flyway. In the population-weighted, each flyway contribution was estimated as its relative contribution to the total population of Anatidae along all flyways multiplied by 100 (100× Popflyway/PopAnatidae). The species included in the analysis are the following: Anas acuta, Anser albifrons, Anser anser, Anser brachyrhynchus, Anas clypeata, Anas crecca, Anser erythropus, Anser fabalis, Aythya ferina, Aythya fuligula, Aythya marila, Aythya nyroca, Anas penelope, Anas platyrhynchos, Anas querquedula, Anas strepera, Branta bernicla, Bucephala clangula, Branta leucopsis, Branta ruficollis, Cygnus columbianus, Cygnus cygnus, Clangula hyemalis, Cygnus olor, Mergellus albellus, Marmaronetta angustirostris, Melanitta fusca, Mergus merganser, Melanitta nigra, Mergus serrator, Netta rufina, Oxyura leucocephala, Polysticta stelleri, Somateria mollissima, Tadorna ferruginea, Tadorna tadorna. The data on HPAI locations were extracted from HPAI H5N1 virus reported presence, as recorded in the FAO Empres-I database (*31*) between July 1, 2005, and January 16, 2006.

## Appendix

**Table A1:** Family and species found to be positive for highly pathogenic avian influenza H5N1 virus as of May 15, 2006

Order/Family/Species	English name*	Source
Anseriformes		
Anatidae		
Aix sponsa	Wood duck	(*1*)
Amazonetta brasiliensis	Brazilian teal	(*1*)
Anas acuta	Northern pintail	(*2*)
Anas bahamensis	White-cheeked pintail	(*1*)
Anas castanea	Chestnut teal	(*1*)
Anas platalea	Red shoveler	(*1*)
Anas platyrhynchos	Mallard	(*2*)
Anas sibilatrix	Chiloe wigeon	(*1*)
Anas versicolor	Silver teal	(*1*)
Anser anser	Greylag goose	(*2*)
Anser erythropus	Lesser white-fronted goose	(*2*)
Anser indicus	Bar-headed goose	(*1**,**2**,**4**,**5*)
Aythya ferina	Pochard	(*2*)
Aythya fuligula	Tufted duck	(*2*)
Aythya marila	Greater scaup	(*2*)
Branta canadensis	Canada goose	(*1**,**2*)
Callonetta leucophrys	Ringed teal	(*1*)
Chenonetta jubata	Maned duck	(*1*)
Coscoroba coscoroba	Coscoroba swan	(*1*)
Cygnus cygnus	Whooper swan	(*2*)
Cygnus melanocoryphus	Black-necked swan	(*1*)
Cygnus olor	Mute swan	(*2*)
Dendrocygna viduata	White-faced whistling duck	(*1*)
Melanitta nigra	Common scooter	(*2*)
Mergellus albellus	Smew	(*2*)
Mergus merganser	Common merganser	(*2*)
Nesochen sandvicensi	Hawaiian goose	(*1*)
Netta peposaca	Rosybill pochard	(*1*)
Netta rufina	Red-crested pochard	(*1*)
Charadriiformes		
Laridae		
Larus argentatus	Herring gull	(*2*)
Larus brunnicephalus	Brown-headed gull	(*4*)
Larus ichthyaetus	Great black-headed gull	(*4*)
Larus ridibundus	Black-headed gull	(*1**–**3*)
Ciconiiformes		
Accipitridae		
Accipiter gentilis	Northern goshawk	(*2*)
Buteo buteo	Common buzzard	(*2*)
Buteo lagopus	Rough-legged hawk	(*2*)
Ardeidae		
Ardea cinerea	Grey heron	(*1**–**3*)
Egretta garzetta	Little egret	(*1**–**3*)
Columbiformes		
Columbidae		
Columba livia	Rock pigeon	(*1**,**3*)
Streptopelia decaocto	Eurasian collared-Dove	(*2*)
Falconiformes		
Falconidae		
Falco peregrinus	Peregrine falcon	(*2**,**3*)
Falco tinnunculus	Common kestrel	(*2*)
Sturnidae		
Acridotheres cristatellus	Crested myna	(*2*)
Gruiformes		
Rallidae		
Fulica americana	America coot	(*2*)
Fulica atra	Eurasian coot	(*2*)
Passeriformes		
Corvidae		
Corvus corone	Carrion crow	(*2*)
Corvus macrorhynchos	Large-billed crow	(*2*)
Corvus pica	Common magpie	(*2*)
Corvus splendes	House crow	(*2*)
Passeridae		
Passer montanus	Tree sparrow	(*1**,**3*)
Zosteropidae		
Zosterops japonicus	Japanese white-eye	(*2*)
Pelecaniformes		
Phalacrocoracidae		
Phalacrocorax carbo	Great cormorant	(*2**,**5*)
Phoenicopteriformes		
Phoenicopteridae		
Phoenicopterus ruber	American flamingo	(*1*)
Podicipediformes		
Podicipedidae		
Podiceps cristatus	Great crested grebe	(*2**,**6*)
Strigiformes		
Strigidae		
Bubo bubo	Eurasian eagle-owl	(*2*)

*English names were obtained from the Wikispecies species directory (http://species.wikimedia.org).
